# Molecular cloning and transcriptional and functional analysis of glycogen synthase kinase-3β in *Haemaphysalis longicornis* (Acari, Ixodidae)

**DOI:** 10.1051/parasite/2019038

**Published:** 2019-07-11

**Authors:** Md. Khalesur Rahman, Myungjo You

**Affiliations:** 1 Laboratory of Veterinary Parasitology, College of Veterinary Medicine and Bio-Safety Research Centre, Chonbuk National University Iksan 54596 Republic of Korea; 2 Department of Microbiology, Faculty of Veterinary and Animal Science, Hajee Mohammad Danesh Science and Technology University Dinajpur 5200 Bangladesh

**Keywords:** RNA interference, Vaccine, GSK-3β, Real-time PCR, Cloning

## Abstract

Glycogen synthase kinase 3 (GSK-3), which belongs to the serine/threonine kinase family, regulates glycogen metabolism, Wnt signaling, hormonal regulation, and embryonic development in many eukaryotes. Here, we cloned a complete open reading frame (ORF) of glycogen synthase kinase 3β (GSK-3β) from *Haemaphysalis longicornis* and characterized its transcriptional and functional status. The ORF of GSK-3β possesses 1242 nucleotides encoding a mature protein of 413 amino acid residues. GSK-3β nucleotide and protein sequences are highly conserved among different vertebrate and invertebrate animals, with identity between 47.8–100% and 63.2–88.7%, respectively. Sequence comparison showed one signature domain between the residues of 51 and 335 amino acids, which was identified as a protein kinase (serine/threonine). RT-PCR showed GSK-3β mRNA present in all developmental stages of *H. longicornis*. Interestingly, a higher transcript level was observed in nymph and 7-day-old eggs compared with others by real-time PCR, indicating a role of GSK-3β in the early stages of life. The functional status of GSK-3β was characterized by RNA interference (RNAi) and caused significant (*p* < 0.05) reduction in feeding and reproduction, as well as an abnormality in eggs and hatching. Taken together, our results suggest that GSK-3β may be an important candidate for a multiple antigen vaccine for controlling the tick population.

## Abstract (fr)

La glycogène synthase kinase 3 (GSK-3), qui appartient à la famille des sérine/thréonine kinase, régule le métabolisme du glycogène, la signalisation Wnt, la régulation hormonale et le développement embryonnaire chez de nombreux eucaryotes. Ici, nous avons cloné un ORF complet (cadre de lecture ouvert) de la glycogène synthase kinase 3β (GSK-3β) d’*Haemaphysalis longicornis* et caractérisé son statut transcriptionnel et fonctionnel. L’ORF de GSK-3β inclut 1242 nucléotides, codant pour une protéine mûre de 413 acides aminés. Les séquences nucléotidiques et protéiques de GSK-3β sont hautement conservées chez différents animaux vertébrés et invertébrés, avec une identité comprise entre 47,8–100 % et 63,2–88,7 %, respectivement. La comparaison de séquence a montré un domaine de signature entre les résidus des acides aminés 51 et 335, qui a été identifié comme une protéine kinase (sérine/thréonine). La RT-PCR a montré que l’ARNm de GSK-3β était présent à tous les stades de développement d’*H. longicornis*. Il est intéressant de noter qu’un taux de transcription supérieur, mesuré par PCR en temps réel, a été observé chez les larves et les œufs de 7 jours par comparaison à d’autres, indiquant un rôle de GSK-3β au début de la vie. L’état fonctionnel de GSK-3β a été caractérisé par une interférence ARN (ARNi) qui a entraîné une réduction significative (*p* < 0,05) de l’alimentation et de la reproduction, ainsi qu’une anomalie des œufs et des éclosions. Pris ensemble, nos résultats suggèrent que la GSK-3β pourrait être un candidat important pour un vaccin à antigènes multiples destiné à contrôler les populations de tiques.

## Introduction

Ticks are ectoparasitic arthropods that have gained attention due to their pathogen-transmitting capability to animals and humans throughout the world [9]. Also, ticks are responsible for significant economic losses in livestock production worldwide. *Haemaphysalis longicornis* is a three-host tick that is distributed throughout the Asian countries, including Korea, Japan, and China, as well as in New Zealand and Australia [23].

Tick control is largely dependent on acaricides, anti-tick vaccines, and tick repellents [4]. Due to environmental hazards and resistance development, the applicability of acaricides and tick repellents is limited. Therefore, development of an anti-tick vaccine is a sound alternative for sustainable control of the tick population as well as for tick-borne diseases and increasing environmental protection [11]. The effectiveness of this approach depends on cloning and characterization of tick molecules that play a role in tick physiology.

The tick digestion process is somewhat peculiar. After blood intake, digestion occurs entirely in the midgut, the only digestive organ in the tick body. Digestion helps to store proteins in the tick midgut and to provide an energy source for embryogenesis and egg production [24, 44]. Therefore, disrupting the protein source and thus the energy supply to tick embryos may be a target for control of tick populations.

Glycogen synthase kinase 3 (GSK-3) is a serine/threonine kinase protein widely conserved among vertebrate and invertebrate species [20, 22]. GSK-3 is a vital enzyme that plays a key role in mammalian glycogen metabolism. GSK-3 occurs as two isoforms, GSK-3α and GSK-3β; both forms play multifunctional roles in glucose metabolism, apoptosis, embryonic development, and gene transcription [1, 39].

Glycogen synthase kinase 3, also known as shaggy/zeste-white 3 (GSK-3β only substitutes for this) in *Drosophila*, has 80–90% similarity with insect and mammalian GSK-3β [1, 5]. GSK-3 regulates the Wnt signaling pathway, resulting in low levels of β catenin in the cytoplasm by phosphorylation. Generally, GSK-3 (α and β) integrates to form a protein complex with Axin and adenomatous polyposis coli (APC) scaffold proteins for ubiquitination and ultimately proteasomal degradation [26, 36]. GSK-3β plays an important role in tumor suppression by the phosphorylation of proto-oncogenes c-Jun, c-Myc, cyclin D1, and b-catenin [12, 42].

Previous studies have suggested that GSK-3 may be an interesting target for antiprotozoal drug development. GSK-3 (α and β) inhibitors have been used successfully for control of *Toxoplasma gondii*, *Trypanosoma brucei*, *Leishmania donovani*, and *Plasmodium falciparum* [14, 38, 40, 46]. However, GSK-3 inhibitors are also used in the treatment of many human diseases such as diabetes, Alzheimer’s disease, psychiatric diseases, and schizophrenia, making it a promising target for new drug development [28, 29, 43]. Although mammals, amphibians, fish and lizards have both GSK-3α and GSK-3β, interestingly GSK-3α is absent in *Rhipicephalus microplus* and birds [2, 34].

Wnt signaling is the major pathway of embryogenesis [7, 33]. GSK-3 is a crucial kinase for this process. There is very little information available about tick GSK-3β and its expression. Among ticks, GSK-3β has been described in *R. microplus* [34], whereas GSK-3β inhibition has been stated to reduce hatching and fertility [16]. Therefore, identification and characterization of GSK-3β in *H. longicornis* will help to achieve a deeper understanding of its role in tick physiology.

## Materials and methods

### Tick rearing and feeding

The Jeju strain of *H. longicornis* (hard tick) has been reared since 2003 in the Laboratory of Veterinary Parasitology, College of Veterinary Medicine and Bio-Safety Research Institute at Chonbuk National University, Iksan, Republic of Korea.

### Sample collection

Initially, 25 unfed adult male and female ticks of *H. longicornis* were placed in a cloth sock attached to the ears of SPF (specific pathogen-free) New Zealand White rabbits (Samtako, Korea), using tape. After five days of feeding, 15 partially engorged females were removed for midgut collection. The other 10 female ticks were collected after spontaneous drop-down and were incubated separately at 25 °C for egg laying. For midgut collection, ticks were held for 1 h at room temperature for removal of host tissue. To prevent surface contamination, ticks were cleaned using distilled water and 70% ethanol. Tick midguts were collected as previously described [41]. Briefly, ticks were attached to clean sterile slides (ventral side down) using liquid paraffin. Dissection was performed under a dissecting microscope (Nikon SMZ-U) using a scalpel fitted with a surgical blade no. 11. Midguts were dissected and washed with ice cold 1 × PBS to remove any contamination and then stored immediately in RNAlater solution (Ambion, Austin, TX, USA) at −70 °C.

### RNA extraction and complementary DNA (cDNA) synthesis

Total RNA was extracted from the collected midguts using a total RNA extraction kit (RiboEx™), according to the manufacturer’s instructions. The RNA concentration was quantified using a NanoDrop™ 2000 spectrophotometer (Thermo Fisher Scientific, Waltham, MA, USA). The sample was then stored at −70 °C. According to the manufacturer’s instructions, the complementary DNA (cDNA) was synthesized from 1 μg total RNA by using a Transcriptor first-strand cDNA synthesis kit (Roche Holding AG, Basel, Switzerland).

### Amplification and cloning of GSK-3β cDNA

The sense primer DG-F1 and anti-sense primer DG-R1 shown in Table 1 were used to amplify the *H. longicornis* GSK-3β sequence from egg cDNA as described previously [15] to identify sea urchin GSK-3β cDNA. RT-PCR was performed using AccuPower^®^ ProFi Taq PCR premix (Bioneer, Daejeon, Korea); the thermal profile was maintained as follows: 95 °C for 5 min, followed by 40 cycles at 95 °C for 20 s, 45 °C for 1 min, and 68 °C for 1 min, with a final extension at 68 °C for 5 min. Amplified PCR product (600 bp) was cloned into an All in One™ vector (BIOFACT™, Daejeon, Korea), according to the manufacturer’s instructions. Nucleotide sequences of the positive clones were sequenced. After a sequence similarity search in NCBI, we performed PCR with the primer extension method to identify the full-length sequence. Primers listed in Table 1 were used for PCR and sequencing. After analyzing all sequences, we identified a complete open reading frame (ORF) of 1242 bp. The nucleotide sequence of GSK-3β described in this paper has been submitted to the GenBank database under accession number MK191019.

**Table 1:** List of Primer used in this experiment.

Primer name	Sequence (5′–3′)	Product size	Use
DG-F1	GTIGCIATHAARAARGTIYTICARGAY	600 bp	For Preliminary identification of GSK-3β
DG-R1	YTTRWRYTCIRTRTARTTIGGRTTCAT		
Orf-F1	ATGAGTGGACGGCCGAGGACG	1248 bp	Primer extension
Orf-R1	AGTGGCCCCGTGTAAATAAAG		
GSK-F1	CGCCTGGACCACTGTAACAT	351 bp	For transcriptional analysis
GSK-R1	CGAGCAGATGTACGACACGT		
Actin-F1	AGCGTGGCTACTCTTTCACC	229 bp	
Actin-R1	GATTCCATACCCAGGAACGA		
T7-GSK-F1	TAATACGACTCACTATAGGGTACTGCGCTTCAAGAACCGCGAA	318 bp	For ds RNA synthesis
T7-GSK-R1	TAATACGACTCACTATAGGGTACTTTTAGTACGCCCGTCTCTGG		

*Note*. The underlined bases denote T7 promoter sequences.

### Sequence analyses

Nucleotides and the deduced amino acid sequence were analyzed using online EMBOSS translation programs (https://www.ebi.ac.uk/Tools/st/emboss_transeq/). Multiple sequence alignment was performed by T-coffee (http://tcoffee.crg.cat/apps/tcoffee/do:regular) [45] combined with BioEdit software (7.2.1) implementing the ClustalW algorithm using the known GSK-3β amino acid sequences of different species from the GenBank database. A phylogenetic tree was constructed with MEGA X software using neighbor-joining methods [31].

### Transcriptional analysis of GSK-3β

Total RNA was extracted from the collected eggs of different laying periods (1, 3, 5, and 7 day-old eggs), different developmental stages (larvae, nymph, and adult), and different adult midgut stages (unfed, fed, and five days after dsRNA injection) using a total RNA extraction kit (RiboEx™), following the manufacturer’s instructions. Complementary DNA (cDNA) was synthesized using a Transcriptor first-strand cDNA synthesis kit (Roche Holding AG, Basel, Switzerland), according to the manufacturer’s instructions, using 1 μg of total RNA from each group. For GSK-3β transcriptional analysis, RT-PCR (conventional PCR) and real-time PCR (quantitative PCR) was performed. RT-PCR was performed as mentioned above. Real-time PCR was performed by using a One-step SYBR^®^ PrimeScript™ RT-PCR kit II (Clontech Laboratories, Mountain View, CA, USA) with a Thermal Cycler Dice™ system (Takara, Kyoto, Japan), according to the manufacturer’s recommendations. The gene-specific primers GSK-F1 (sense) and GSK-R1 (anti-sense) were used for the transcriptional analysis shown in Table 1. Data were normalized with an internal control actin (GenBank AY254898.1), and the ΔΔ*Ct* value was calculated as described elsewhere [35].

### Generation and injection of GSK-3β double-stranded RNA (dsRNA)

The PCR products of GSK-3β (318 bp) were joined to a T7 promoter sequence on both sides (5′ and 3′). A T7 promoter sequence was added, as described in our previous experiment [41] by using a gene-specific and T7 promoter-linked primer T7-GSK-F1 (sense) and T7-GSK-R1 (anti-sense), shown in Table 1. A HiScribe™ T7 High Yield RNA Synthesis Kit (New England Biolabs, Inc., Hitchin, UK) was used to synthesize dsRNA, according to the manufacturer’s protocol. The dsRNA was quantified by a NanoDrop™ 2000 spectrophotometer (Thermo Fisher Scientific, Waltham, MA, USA). Injection of GSK-3β dsRNA (approximately 0.5 μL of 1 μg/μL dsRNA) was performed as described elsewhere [41]. The control group was injected with unrelated dsRNA (Luciferase dsRNA). After injection, the ticks were kept overnight in a 25 °C incubator with high humidity to observe their survival. Then, injected ticks were allowed to feed with male ticks on the ears of an SPF rabbit. To evaluate gene silencing, 12 female ticks were collected from both groups after five days of attachment. Their midguts were subsequently collected, and real-time PCR was performed for gene knock-down analysis. Twelve additional dsRNA injected ticks were fed to spontaneous drop-down. The tick attachment rate, feeding and oviposition period, blood engorgement, egg mass, and egg abnormality and hatchability were recorded.

### Statistical analysis

Statistical analysis was performed by Graph Pad Prism 5 (GraphPad Software, Inc., La Jolla, CA, USA) using Student’s *t*-test (unpaired and unequal variances) and One-way ANOVA with Bonferroni’s multiple comparison tests. Values are presented as mean (*M*) ± standard deviation (*SD*). A *p*-value of ≤ 0.05 was considered significant.

## Results

### Cloning and sequence analysis of the partial cDNA encoding *H. longicornis* GSK-3β

**Figure 1:**
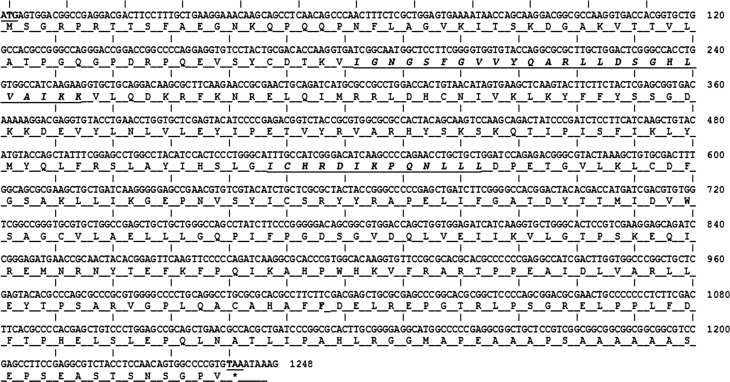
GSK-3β nucleotide sequence from *Haemaphysalis longicornis* and its deduced amino acid sequence. Numbers of the nucleotide sequence are on the right side. One protein kinase domain was found between amino acid residues 55 and 339. ATP binding sites located between residues 61 and 85 are shown in underlined bold letters. The serine/threonine protein kinase active site located between residues 176 and 188 is also shown in underlined bold letters. Start codon and stop codon are highlighted with underlined bold letters.

GSK-3 is a crucial enzyme responsible for phosphorylation of many enzymes in eukaryotic cells [27]. Here, we identified a complete ORF of GSK-3β cDNA from *H. longicornis* extending from 1 to 1242 bp by PCR and cloning. PCR amplification was performed using the degenerate primer method, as described previously [15]. Nucleotide sequences were translated to corresponding amino acid sequences using EMBOSS translation programs (https://www.ebi.ac.uk/Tools/st/emboss_transeq/) and represented 413 amino acids. The theoretical molecular mass was 45937.88 Da, and the pI was 8.88 as detected by ExPASy, the Molecular Biology Server of the Swiss Institute of Bioinformatics (https://www.expasy.org/). Interpro analysis (http://www.ebi.ac.uk/interpro/search/sequence-search) on amino acid sequences identified an ATP binding region of the protein kinase between residues 61 and 85 and a protein kinase signature domain between residues 55 and 339. An active serine/threonine protein kinase site was identified between residues 176 and 188, and a glycogen synthase kinase-3 catalytic domain was found between residues 50 and 342 (Fig. 1). The GSK-3β amino acid sequences were analyzed by NJ with Poisson corrections and 500 bootstrap replicates by MEGA-X software (Fig. 3).

### Sequence similarity and phylogenetic analyses

**Figure 2:**
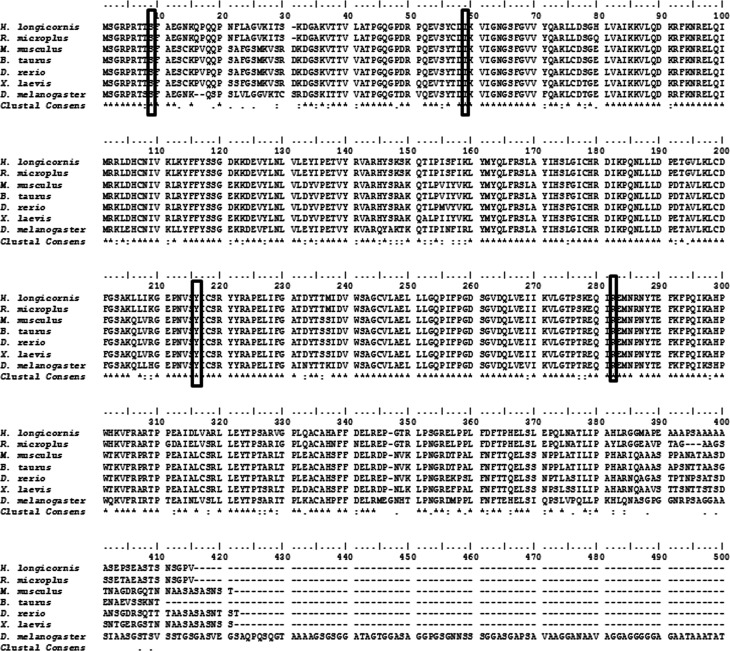
Multiple sequence alignment of deduced GSK-3β amino acid sequences from different species. Asterisks indicate conserved residues. Conserved GSK-3β active sites are indicated in boxed letters (N-terminal conserved glycine, amino acids 59 and 282), as are conserved phosphorylation sites of serine and threonine (amino acid 9 and 216, respectively).

Our 1248-bp cloned sequence and corresponding 413-amino acid sequence were compared with other known GSK-3β sequences from the NCBI database using sequence alignment software T-coffee (http://tcoffee.crg.cat/apps/tcoffee/do:regular). Alignment showed that the conserved active GSK-3β sites were located in the amino acid sequence at 59 and 282 (N-terminal conserved glycine). The position of the N- terminal serine and tyrosine residue at amino acid sequence nos. 9 and 216, respectively indicated the identity of GSK-3β (Fig. 2).

GSK-3β amino acids and nucleotide sequences from different species (*R. microplus* ABO61882.1, *Drosophila melanogaster* AAM52705, *Mus musculus* AAH60743, *Bos taurus* XP878051, *Xenopus laevis* AAC42224, *Danio rerio* NP_571456, *Rattus norvegicus* NP_114469.1, *Ovis aries* NP_001123212.1, *Gallus gallus* XP_416557.4, *Aedes albopictus* XP_019545260.1, *Tetranychus urticae* XP_015794496.1, *Tribolium castaneum* XP_015834727.1 and *Plasmodium falciparum* XP_015834727.1) were collected from the NCBI database and compared for identity with our *H. longicornis* GSK-3β sequence. Identity percentages of GSK-3β nucleotides and amino acids sequences from different species are shown in Table 2.

**Table 2:** The percentage identity of amino acid (lower diagonal italics letter) and nucleotide (higher diagonal bold letter) sequences of GSK-3β *H. longicornis* and different species.

	*H. longicornis*	*R. microplus*	*D. melanogaster*	*X. laevis*	*M. musculus*	*D. rerio*	*B. taurus*
*H. longicornis*	100	**88.3**	**70**	**75.1**	**69.2**	**70.9**	**69**
*R. microplus*	*94.4*	100	**69.3**	**70.5**	**65.3**	**69.6**	**67.1**
*D. melanogaster*	*76.7*	*76.6*	*100*	**65.3**	**63.2**	**66.2**	**67.6**
*X. laevis*	*76.4*	*76.4*	*72.9*	100	**78.4**	**80.0**	**78.4**
*M. musculus*	*79.1*	*77.6*	*74.6*	*92.9*	100	**75.1**	**88.7**
*D. rerio*	*77.9*	*76.6*	*74.5*	*91.9*	*94.0*	100	**75.0**
*B. taurus*	*77.9*	*76.9*	*76.2*	*93.8*	*98.8*	*93.6*	100
*R. norvegicus*	*79.1*	*77.8*	*76.4*	*92.9*	*100*	*94*	98.8
*O. aries*	*76.9*	*77.3*	*74.1*	*92.9*	*98.6*	*93.9*	99.3
*G. gallus*	*77*	*75.4*	*71.6*	*90.2*	*93.6*	*92.4*	93.6
*A. albopictus*	*78.2*	*80.3*	*74.9*	*77.1*	*77.1*	*77.9*	78
*T. urticae*	*75.3*	*76.8*	*68.9*	*73.8*	*69.3*	*69.5*	73.9
*P. falciparum*	*55.3*	*54.7*	*56.3*	*47.8*	*48.5*	*49.1*	53.8
*T. castaneum*	*78.3*	*78.2*	*73.5*	*74*	*73.9*	*73.5*	75.6

**Figure 3:**
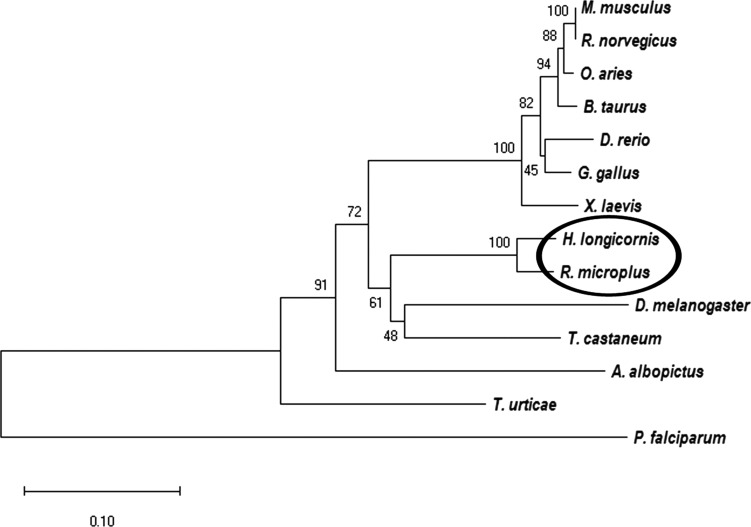
Phylogenetic analysis of GSK-3β from *Haemaphysalis longicornis*. Bootstrap proportions are indicated at branches. Sequences with NJ involving Poisson corrections and bootstrap analysis of 500 replicates. *Haemaphysalis longicornis* and *R. microplus* are in the same clade.

Phylogenetic analysis of GSK-3β sequences was conducted using amino acid sequences from different species (mentioned above) collected from the NCBI database. The results were analyzed by NJ with Poisson corrections and 500 bootstrap replicates by MEGA-X software (Fig. 3).

### Transcriptional analysis of GSK-3β in ticks at different developmental stages by RT-PCR and real-time PCR

**Figure 4:**
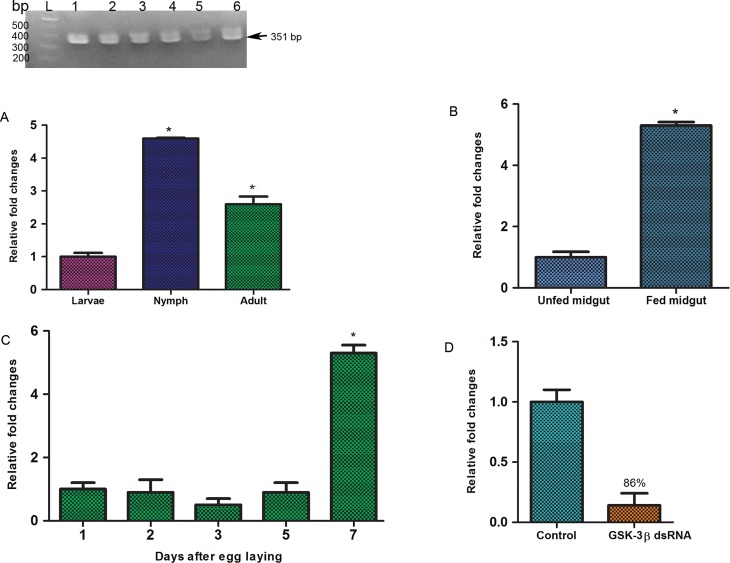
Transcriptional profiles of GSK-3β at different developmental stages. (A) RT-PCR shows presence of GSK-3β in all developmental stages. Lane L indicates 100 bp DNA ladder; lanes 1, 2, 3, 4, 5 and 6 represent GSK-3β present in egg, larva, nymph, adult, unfed midgut and fed midgut, respectively; (B) GSK-3β expression in larva, nymph, and adult stages; (C) GSK-3β expression in fed and unfed midguts of adult females; (D) Expression at different egg ages; (E) Knockdown of GSK-3β compared with the control group. The asterisk (*) denotes a significant difference compared with the control group as determined by Student’s *t*-test (*p* < 0.05) in case of B and D and One-way ANOVA with Bonferroni’s multiple comparison tests in case of A and C (*p* < 0.05).

The life cycle of the *H. longicornis* tick consists of four life stages: egg, larva, nymph, and adult. The blood feeding capability of larvae, nymphs, and adults is different from one stage to another. For transcriptional analysis of GSK-3β, total RNA from different life stages (egg, larva, nymph, and adult) and the adult midgut under different feeding conditions were subjected to RT-PCR and real-time PCR. GSK-3β mRNA transcripts were detected in all developmental stages (Fig. 4A). The degree of transcriptional status was measured by real-time PCR. In the nymph stage, GSK-3β mRNA transcription was relatively higher than in other stages of development (Fig. 4B). The expression level of GSK-3β mRNA in nymphs and adults was 4.6- and 2.6-fold higher, respectively, in comparison with larvae. Interestingly, in adult ticks after a blood meal, expression levels of GSK-3β mRNA in the midgut were significantly (5.3-fold) higher than in the unfed midgut (Fig. 4C). 7-day-old eggs showed significantly higher transcriptional status compared with freshly laid eggs (Fig. 4D).

### GSK-3β knockdown and its effects

Unfed adult female ticks were injected with GSK-3β or unrelated dsRNA and allowed to feed on a rabbit’s ear with an equal number of male ticks. GSK-3β knockdown was confirmed by real-time PCR. After four days of attachment, expression of GSK-3β dsRNA-injected ticks was 86% silenced compared with control ticks (Fig. 4E).

**Figure 5:**
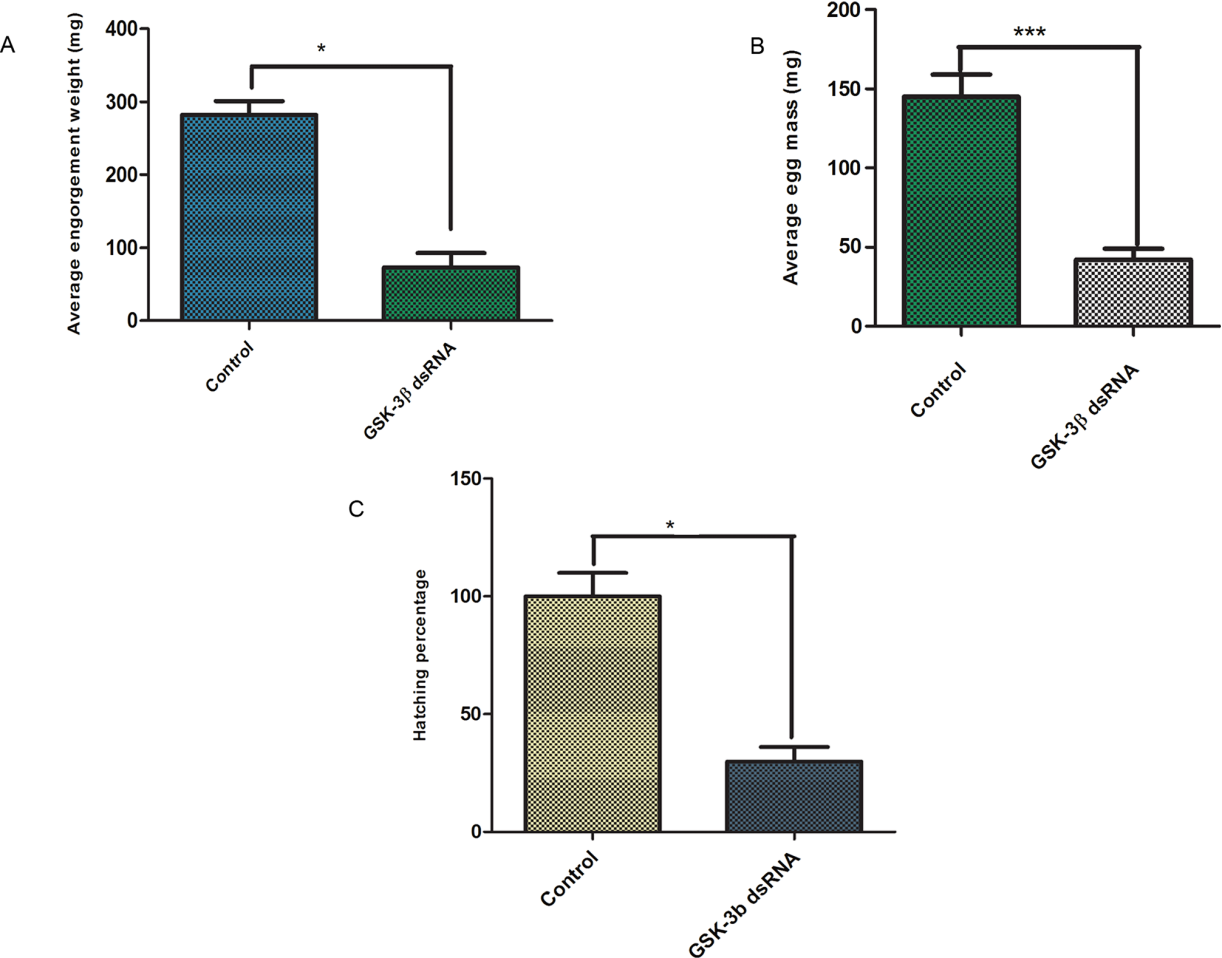
Knockdown effects of GSK-3β dsRNA on (A) tick engorgement, (B) and (C) reproduction. The asterisk (*) denotes a significant difference compared with the control group as determined by Student’s *t*-test (***p < 0.001).

The effects of GSK-3β knockdown on engorgement weight were analyzed after self-drop-down of the injected ticks. GSK-3β knockdown caused significant (*p* < 0.0001, df4) loss of engorgement weight in comparison with the control group (unrelated dsRNA-injected group). The average tick engorgement weight in the GSK-3β dsRNA-injected group was 73.2 mg, while that in the control group was 282 mg (Fig. 5A). However, there was no signifacant variation concerning attachment rates (100%), feeding (6–7 days), and oviposition period (9–10 days) between the control and treatment groups after silencing of GSK-3β.

**Figure 6:**
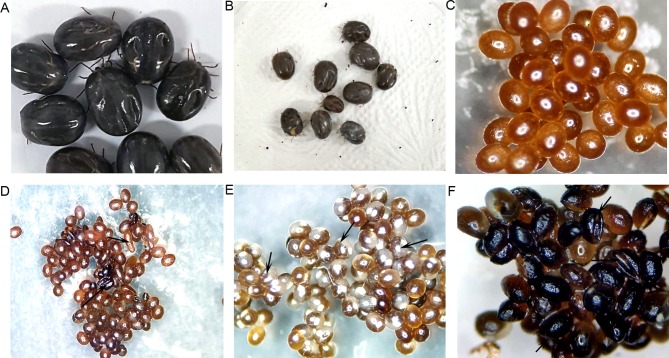
Phenotypic changes in tick (*Haemaphysalis longicornis)* engorgement and egg morphology after GSK-3β dsRNA treatment. (A) Control ticks; (B) GSK-3β dsRNA-treated ticks after spontaneous drop-down; (C) Control tick eggs; (D) Abnormal eggs of GSK-3β dsRNA-treated ticks; (E) Control tick eggs containing embryos; (F) Abnormal eggs (some have no embryos).

To determine the effects of GSK-3β knockdown on reproduction, we recorded egg mass weight, egg abnormality, and hatchability after spontaneous drop-down of dsRNA-injected ticks. GSK-3β dsRNA caused significant reduction of egg production (*p* < 0.0027, df4) and egg hatchability (*p* < 0.0001, df8) compared with the control group (Fig. 5B and C, respectively), egg abnormalities were visible in GSK-3β dsRNA-treated ticks (Fig. 6C–F). Phenotypic changes are shown in Figure 6A and B.

## Discussion

Although ticks are important vectors related to disease transmission [47], our knowledge about their molecular and functional materials are far from that of insects and other species. To control the tick population, identification and characterization of new antigens are promising steps. Molecular cloning and transcriptional analysis will help identify new antigens and their roles in tick physiology. In this context, GSK-3 is an interesting target as it is highly conserved and plays multi-functional roles in different species [13].

GSK-3β has a potential role in tick control. Fabres et al. [16] found that silencing of GSK-3β causes significant reductions in oviposition and hatching. To date, no GSK-3β has been characterized and no tests on its silencing effects in *H. longicornis* have been performed. In this study, we cloned and sequenced a complete ORF of GSK-3β from *H. longicornis.* Analysis of nucleotide and deduced amino acid sequences of GSK-3β showed that it is highly conserved among other known vertebrate and invertebrate species. GSK-3β amino acid sequences were compared using a pair-wise identity measurement program (https://embnet.vital-it.ch/cgi-bin/LALIGN) [25]. Results showed that the putative GSK-3β amino acid sequence from *H. longicornis* shares 94.4% identity with that of *R. microplus* and 76.4–79.1% identities with *D. melanogaster*, *X. laevis*, *M. musculus*, *D. rerio*, and *B. taurus* (Table 2). These results suggest that the cloned gene was the GSK-3β of *H. longicornis.*

Amino acid sequence alignment of different species showed that all compared sequences (vertebrate and invertebrate) share major signature domain and active sites (Fig. 2). These signature domains include a protein kinase (residues 55–339), a serine/threonine kinase (residues 176–188), and a GSK catalytic domain (residues 50–342), as described elsewhere [34]. The position of serine and tyrosine residues at 9 and 216, respectively are highly conserved among different species and regulate the function of GSK-3β by the phosphorylation procedure [17, 32]. For a better understanding of the relationships of GSK-3β amino acid sequences from 14 different species, we generated a phylogenetic tree (Fig. 3). The phylogenetic tree results suggest that *H. longicornis* and *R. microplus* GSK-3β are in the same clade, separate from mammalian and other GSK-3βs.

One previous study showed that GSK-3 plays a key role as an enzyme in the Wnt signaling pathway of vertebrate and invertebrate animals [18], and that this pathway is ultimately necessary for adult tissues [21]. Wnt signaling is required for *Xenopus* and *Drosophila* axis development and segment polarity. In the tick, the embryonic stage is very sensitive for its developments; therefore, researchers have been focused on this stage for the development of anti-tick control strategies [8]. As an important protein, GSK-3β plays a major role in early developmental stages [16, 34]. During the early developmental stage, the midgut, fat body as well as the ovary of arthropods synthesize fat to meet energy demands. Thus, we performed transcriptional analyses of GSK-3β at different developmental stages, different statuses of the midgut, and different ages of eggs. The GSK-3β transcription status was significantly higher in nymphs than in other stages. This is likely because ticks are more active in the nymph stage and need more energy. Findings also support the main function of GSK-3 in glucose metabolism [30]. Additionally, our results align with the conclusions of Logullo et al. [34], who also reported that the midgut of partially fed ticks showed higher GSK-3β transcriptional levels compared with unfed ticks.

Oviparous animal embryos depend on internal nutrients for development before hatching. A recent study on *R. microplus* embryogenesis showed that 21 days are needed to complete embryonic development. The syncytium develops four days after oviposition; the blastoderm appears at day 6 and segmentation at day 7. Embryos need large amounts of nutrients to meet the energy demands of segmentation [6]. This result also supports our present finding that higher GSK-3β transcription was observed in 7-day-old eggs (Fig. 4C).

Furthermore, to determine the functional role of GSK-3β, we conducted an RNA interference experiment. RNA interference is a relatively new molecular technique first introduced by Fire et al. [19]; however, it is widely applied to functional gene analysis in different species [3]. RNA interference acts by degrading targeted mRNA as a result of specific gene inhibition [37]. Injection of GSK-3β dsRNA in adult ticks caused 86% silencing of GSK-3β expression at the mRNA level (Fig. 4D). A major impact of GSK-3β knockdown was found on blood engorgement, abnormal egg production and hatchability (Fig. 6). Taken together, these results suggest that GSK-3β has important roles in embryonic development. Similar effects have been obtained by knockdown of subolesin in different tick species [10, 35].

In summary, we identified cDNA encoding a complete ORF of GSK-3β from *H. longicornis* for the first time. GSK-3β is present in all developmental stages, though expression in embryonic stages seems to be most important. Silencing of GSK-3β affects the feeding and reproductive capabilities of ticks. Further studies are required for recombinant GSK-3β production and assessment of its vaccine candidature for the control of tick populations.
